# How Do Chinese People View Cyberbullying? A Text Analysis Based on Social Media

**DOI:** 10.3390/ijerph19031822

**Published:** 2022-02-05

**Authors:** Shan Lu, Lingbo Zhao, Lizu Lai, Congrong Shi, Wanyue Jiang

**Affiliations:** 1 Key Laboratory of Adolescent Cyberpsychology and Behavior, Ministry of Education, Wuhan 430056, China; lushan@mails.ccnu.edu.cn (S.L.); lynndazu@mails.ccnu.edu.cn (L.L.); psyriver@163.com (C.S.); jwy@mails.ccnu.edu.cn (W.J.); 2 Key Laboratory of Human Development and Mental Health of Hubei Province, Wuhan 430056, China; 3 School of Psychology, Central China Normal University, Wuhan 430056, China; 4 Department of Applied Psychology, School of Humanities and Social Sciences, Fuzhou University, Fuzhou 350108, China

**Keywords:** cyberbullying, attitude, topic model, sentiment analysis, social media, text analysis

## Abstract

The rise of cyberbullying has been of great concern for the general public. This study aims to explore public attitudes towards cyberbullying on Chinese social media. Cognition and emotion are important components of attitude, and this study innovatively used text analysis to extract the cognition and emotion of the posts. We used a web crawler to collect 53,526 posts related to cyberbullying in Chinese on Sina Weibo in a month, where emotions were detected using the software “Text Mind”, a Chinese linguistic psychological text analysis system, and the content analysis was performed using the Latent Dirichlet Allocation topic model. Sentiment analysis showed the frequency of negative emotion words was the highest in the posts; the frequency of anger, anxiety, and sadness words decreased in turn. The topic model analysis identified three common topics about cyberbullying: critiques on cyberbullying and support for its victims, rational expressions of anger and celebrity worship, and calls for further control. In summary, this study quantitatively reveals the negative attitudes of the Chinese public toward cyberbullying and conveys specific public concerns via three common topics. This will help us to better understand the demands of the Chinese public so that targeted support can be proposed to curb cyberbullying.

## 1. Introduction

Today, the Internet is highly developed. We can share all kinds of information with people from all over the world through the Internet, as well as our opinions on social media. However, while the Internet has brought convenience, cyberbullying has become common through the accessibility of electronic media. Cyberbullying is any behavior performed through electronic or digital media by individuals or groups that repeatedly communicate hostile or aggressive messages with the intention of inflicting harm or discomfort on others [1]. Anyone can easily harm and bully other users in electronic environments such as social media by posting malicious comments about another person and spreading rumors about others. Cyberbullying often leads to various consequences, ranging from emotional reactions such as anger, depression, and anxiety, to social difficulties and even suicide [2].

Previous studies have reported the prevalence of cyberbullying in different groups with inconsistent findings. A comprehensive review involving 11 countries showed that the victimization and perpetration rates of cyberbullying among adolescents and children ranged from 14.6% to 52.2% and 6.3% to 32%, respectively, with the highest perpetration rate of 46.3% in China [3]. Among adults, the overall prevalence of cyberbullying is 14.9%, with older age cohorts less likely to experience cyberbullying, but with 40.5% of young adults reporting having experienced cyberbullying [4]. Additionally, one study even pointed out that 64.3% of college students reported that they suffered from cyberbullying attacks [5].

The inconsistency in the prevalence of cyberbullying may be related to the different understandings of cyberbullying. Perception varies depending on the role (e.g., bullies, victims, and bully−victims) and the context of the bullying experience [6]. Students’ moral judgments and emotions of cyberbullying also vary depending on their age, cyberbullying experiences, and thinking perspectives [7]. Many minority and low-income youth are less likely to use the term "cyberbullying", and assume that cyberbullying only occurs when it leads to suicide or severe depression [8]. Thus, the public cognition of cyberbullying is not always as clear as researchers have defined it. This implies that there is a lot of uncertainty about how exactly the public perceives cyberbullying, that is, their unclear attitude towards it.

### 1.1. Attitude towards Cyberbullying

Attitude is important in cyberbullying. According to the Barlett Gentile cyberbullying model [9], a positive attitude toward cyberbullying can directly predict behavior, and the strength of this relationship is stronger than the relationship between attitudes toward traditional bullying and behavior. Some intervention programs have been designed to prevent cyberbullying by reducing positive attitudes or reinforcing negative ones, e.g., the theory of a reasoned action-based video program [10]. In short, clarifying attitudes can help supplement the background of cyberbullying and make relevant policies and interventional programs work better.

Most studies have shown people’s negative attitudes towards cyberbullying. A survey found that 89.4 % of adolescents held a negative attitude towards cyberbullying [11]. In addition, a recent cross-cultural study measured attitudes toward cyberbullying in seven countries including Australia, Brazil, China, Germany, Japan, Singapore, and the United States, and presented that positive attitudes toward cyberbullying were quite low across countries, with China scoring the highest on average [12]. However, some other evidence has shown that attitudes towards cyberbullying are likely to be mixed. Munnelly et al., for the first time, conducted an implicit relational assessment procedure and self-report measures to examine adolescents’ implicit and explicit attitudes toward cyberbullying [13]. It was found that adolescents displayed a combination of anti- and pro-cyberbullying explicit attitudes, while their implicit attitudes were completely anti-cyberbullying. Adolescents’ anti-cyberbullying attitudes were not so positive, and most of them had neutral or even negative anti-bullying behavior intentions [14].

The three components of attitude that influence each other are cognition, emotion, and behavioral tendency. Note that current techniques can measure emotion and cognition well, but it is difficult to measure behavioral tendency directly because behavioral tendency is a reaction preparation state before a specific action. Van Aalderen-Smeets et al. argued that behavior should be removed from the attitude model because they found that few attitude studies contained components that could be categorized as behaviors or behavioral intentions [15]. Some scholars were even more supportive of the two-component affect−cognition model of attitude [16]. Current attitude research is primarily focused on emotion and cognition.

### 1.2. Exploring Attitude with Natural Language Processing (NLP) Techniques

Previous studies have used traditional research methods, such as questionnaires, interviews, and experiments, to describe one component of attitudes. However, few studies have been able to analyze people’s attitudes to cyberbullying on multiple components simultaneously. Moreover, traditional research methods are weak in sample size, ecological validity, data objectivity, and so on. Fortunately, technological advances have opened up the possibility of methodological innovation in the field of exploring attitudes toward cyberbullying.

NLP techniques can easily analyze the emotional and cognitive components of attitudes from textual material [17]. We are so excited about the contribution of textual material to exploring attitudes. This means that we can track people’s opinions based on the vast amount of text that exists on the Internet and social media. The Internet and social media play an important role in defining a psychological profile [18,19]. People’s mental states from what they write on Facebook, Twitter, and other social media can be inferred clearly with NLP techniques [20]. Various studies have supported this; for example, analyzing people’s social media accounts can provide insight into their suicidal intentions [21] and even their attitudes toward vaccines [22]. Public statements on social media convey information about public attitudes and make a difference in influencing others [23]. Therefore, analyzing people’s discourse using NLP techniques, combined with the analysis of linguistic features and semantic structure, may be a better way to explore the cognitive, emotional, and behavioral aspects of attitudes [24].

For the emotional and cognitive components of attitude, the suitable NLP techniques are sentiment analysis and content analysis techniques.

Sentiment analysis automatically extracts subjective information from the text and assesses whether someone’s view on something is negative, positive, or neutral [25]. Many methods of sentiment analysis have developed [26,27] that can identify more emotions. The preferred automated sentiment analysis method in psychology is the linguistic inquiry and word count (LIWC) [28]. Its core is a dictionary that has 80 categories of words with psychological meaning [28]. Among them, emotion words are classified into two categories—positive emotion and negative emotion—with negative emotion including three subcategories: anxiety, anger, and sadness [28]. LIWC has been shown to predict emotions relatively accurately [28,29,30,31], and is widely used in attitude research [28,30,32]. These five emotions are common in research and everyday expression. Positive and negative emotions are two key dimensions of emotions that determine the affective polarity of attitude [33], whereas studies have demonstrated that negative emotions have a greater impact on attitudes [34], so they are worthy of further analysis. The three subcategories of negative emotion words, in addition to being commonly used and having psychological research meaning, have the advantage of being sensitive to changes in attitudes with different contexts [24,35]. Thus, we are concerned with their role in attitude formation.

Content analysis techniques are rich in a variety of ways that aim to discover topics of a text [36]. Among them, the Latent Dirichlet Allocation (LDA) topic model [37] is widely used in many fields and is easy to understand. The topic model is developed from text analysis techniques based on word frequency. It is a Bayesian probability model with word, topic, and document levels. This statistical model uses unsupervised machine learning to find latent semantic structures in a series of documents and automatically generates topics. Recently, studies on Chinese social media, such as the attitudes toward depression, have used the LDA topic model to analyze posts on Sina Weibo, a social media platform known as the Chinese version of Twitter, in order to deduce public attitudes [24].

As we all know, cyberbullying occurs in different online channels, especially on social media sites [38]. Users of social media, who have more chance of encountering cyberbullying, play a key role in preventing this international problem. Exploring their attitudes would be very helpful for guiding investigation and intervention research in the future. In recent years, some studies have used social media data from Twitter users to analyze attitudes toward cyberbullying. McHugh et al. collected English posts, searched with the keywords of cyberbullying over a month, and applied LIWC and a hand-coded method to analyze the sentiment and content of the posts, respectively [32]. They found that most people hold a neutral attitude when it comes to discussing cyberbullying, and they love to talk about the cyberbullying situation. However, another study shows that Twitter users mainly have a negative attitude toward cyberbullying. Tahamtan and Huang used correlation network analysis and Bing’s lexicon approach to explore topics and emotions in the discussion about cyberbullying, and found that people mentioned more negative words and discussed more cyberbullying prevention [39].

### 1.3. The Current Study

On social media, attitudes towards cyberbullying among English-speaking users, most of whom come from the US and Europe, have been explored. However, few studies have explored the attitudes among Chinese users. There are differences across cultures in various aspects of cyberbullying [40], including attitudes toward cyberbullying [12]. Thus, it is valuable to investigate users’ attitudes towards cyberbullying from the perspective of cultural differences. Sentiment and content analysis techniques offer new ways to understand public attitudes. To expand the findings on attitudes toward cyberbullying, this study aims to analyze public attitudes on social media in Chinese culture through a more advanced approach.

Given our proposed research objective, this study will use LIWC and LDA topic model techniques to analyze masses of posts from users on Sina Weibo, the most popular social media platform in China. We expect that, like most previous studies, when the Chinese public refers to cyberbullying, they are likely to have a more negative emotion than positive emotion (hypothesis 1). The content analysis will reveal some common topics that are likely to be different from those in other cultural contexts (hypothesis 2). This study will further crystallize public attitudes in terms of their emotion and cognition in light of big data. This helps to understand common opinions, propose targeted support to meet the demands of the Chinese public, and pave the way for cyberbullying prevention and intervention.

## 2. Materials and Methods

### 2.1. Data Collection

Sina Weibo is a widely used platform in China, with more than 550 million monthly active users, where people often express their opinions publicly. The platform supports advanced search, which allows users to search for specific keywords, define a range of dates, and send requests for obtaining posts that contain qualified characters. Text analysis studies of public attitudes often collect posts posted over a period of several days [39] or even years [24]. Following the approach in McHugh et al. [32], we selected Weibo posts posted over a single month to analyze attitude.

Using the function of advanced search, our study collected posts related to cyberbullying from 20 February 2020 to 20 March 2020, which were open to everybody and easily accessible. As we did not invade any individuals’ privacy by disclosing users’ identities, there were no ethical issues to address. We used the Selenium web crawler tool [41] to search for posts that contained predefined keywords such as “cyberbully”, and obtained 69,141 Weibo posts. Due to the instability of the Internet, some of these posts were invalid and contained garbled codes or outliers. Additionally, there were also some duplicate and reposted posts. After we cleaned up these posts that did not meet our requirements, 53,526 posts remained, which entered the subsequent analysis.

### 2.2. Data Processing

All the content analyses were conducted by Python 3.8. As Chinese sentences have no obvious spaces between words, it is necessary to segment the words of the texts with a space in advance so as to complete the processing tasks of the LDA topic model. A Chinese word segmentation toolkit, Jieba [42], was applied to divide Weibo posts into groups of words separated by space.

Then, we removed the stopwords. The stopword list was primarily based on several widely used Chinese stopword lists [43] and was supplemented by words such as “one”, “because”, and “first”, according to the results of multiple word segmentation trials. Non-Chinese characters such as English characters, punctuation, emojis, and numbers were also deleted. In addition, we eliminated the search keyword of “cyberbully”, which appeared in all posts collected and could not provide more information for content analysis [44].

### 2.3. Data Analysis

First, we conducted a sentiment analysis. Gao [45] established a Simplified Chinese LIWC (SC-LIWC) dictionary according to the classification of LIWC and added the most commonly used words in Sina Weibo to it. "Text Mind" [46] is a Chinese linguistic psychoanalysis software, taking SC-LIWC as a built-in dictionary. It can easily and automatically label psychological linguistic categories for words in the text. Like LIWC, it has two emotional linguistic categories, including positive emotion and negative emotion, and three subcategories of anxiety, anger, and sadness (Table 1). When "Text Mind" runs, it outputs the frequency of words of each emotional category out of the total number of words in each post. The larger the frequency, the stronger the post possesses this emotion. In this way, the degree of emotion expressed in this post can be quantified [28,29]. For example, if "Text Mind" analyzes a text containing 1000 words, it will compare each word with a built-in dictionary, and may find that the text possesses 45 words of anger and 110 words of negative emotion, which leads to a frequency output of 0.045 and 0.11 for anger and positive emotion, respectively.

Second, we conducted a content analysis. Topic model is a machine learning method for discovering abstract topics in a series of texts. The LDA topic model [37] is the first complete probabilistic semantic generation model. It is a statistical model that uses unsupervised machine learning to discover latent semantic structures in a series of texts. The LDA topic model treats the document as a “bag of words” and represents it as a vector, transforming text into easily processed digital information. The LDA topic model can reflect the document−topic−word distributions, identify topics of text, and return words sorted by probability of occurrence under each topic. A topic would be explained by a group of highly co-occurrent terms. The model works well on short texts [48]. We treated each post as a document and used a Python library, Gensim [49]. We set the parameters of 1–30 for the number of topics. Finally, we determined the optimal number of topics by calculating the document-based topic coherence [50] and visualized topical similarity [51] for each number of topics.

After sentiment analysis and content analysis of the entire dataset, each post was given the word frequency of various emotions and the probability that it belonged to each topic. If a post has the highest probability of belonging to a topic, then the post is classified as that topic. Finally, we used SPSS 20.0 to describe the frequency of emotion linguistic features and the classification result of the topic model analysis.

## 3. Results

### 3.1. Sentiment Analysis

When the frequency of an emotional linguistic feature is 0, it means that the post does not contain the emotion. If a post does not contain positive or negative emotion, it is considered as an expression of a neutral attitude. Among all the posts (*n* = 53,526), 10.8% (*n* = 5,781) were positive, 24.3% (*n* = 13,036) were negative, 13.2% (*n* = 7,044) were neutral, and the rest (*n* = 27,665, 51.7%) had both positive and negative emotions.

**Figure 1:**
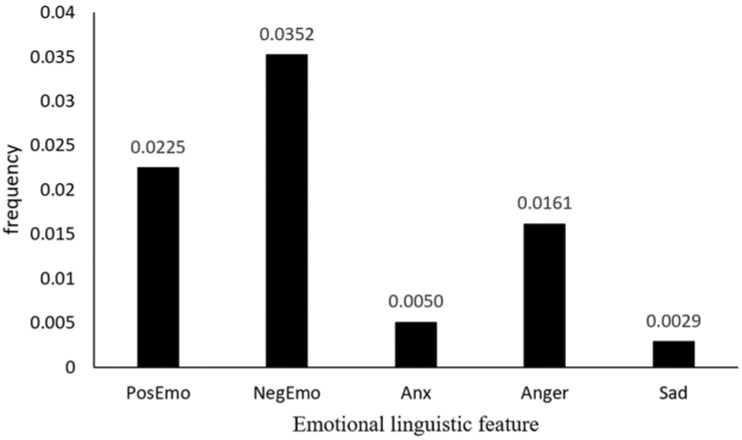
Frequency of five emotional linguistic features of all posts related to cyberbullying (*n* = 53,526). The Y-axis refers to the average frequency of each category (i.e., the number of words in each category divided by the total number of words) appearing in a post; PosEmo = positive emotion; NegEmo = negative emotion; Anx = anxious emotion; Anger = angry emotion; Sad = sad emotion.

We calculated “the sum of the emotional linguistic feature frequency of all posts/53,526” as the average emotional feature values, representing the intensity of the emotion (Figure 1). As you can see, negative emotion dominated. We further analyzed the subset of negative emotion—anxiety, anger, and sadness. Anger was the most common negative emotion, with an average frequency of 0.0161, and 51.84% of posts contained anger. The second was anxiety, with an average frequency of 0.0050, and 20.42% of posts contained anxiety. Finally, sadness was found in 16.72% of posts, with an average frequency of 0.0029.

### 3.2. Cyberbullying Related Topics

**Figure 2:**
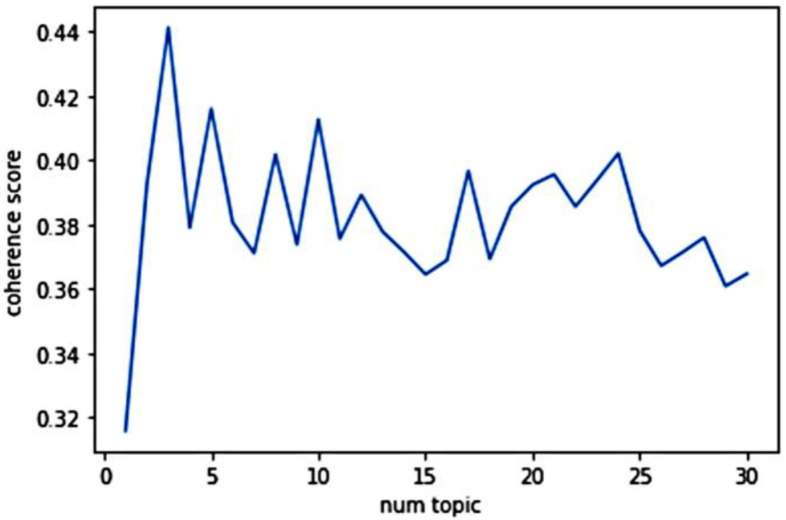
Coherence score for the number of topics. Num topic = the number of topics; coherence score = the coherence score returned by the LDA model.

Common words were generated and organized into different topics by the automated machine learning LDA approach. The document-based topic coherence method [50] was used to calculate the most appropriate number of topics. Figure 2 showed the coherence score for the number of topics, which is returned by the LDA model. The higher the score, the better the quality of generated topics. Therefore, for this dataset, the most appropriate number of topics returned by the LDA model was three.

**Figure 3:**
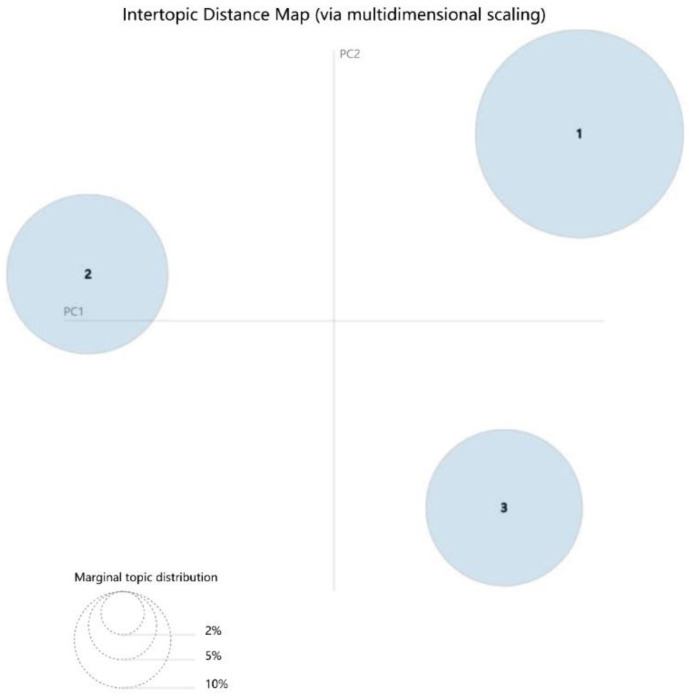
Intertopic distance map. PC = principal component. The circled area is the overall prevalence, and the center of the circle is determined by computing the distance between topics.

In addition, the intertopic distance map was drawn on a 2D plane (Figure 3) [51]. Each circle represents a topic from Topic 1 to Topic 3 in the study. The centers are determined by computing the distance between topics. In the visualization, these circles do not overlap, and thus the classification of the three topics was cross-validated.

The document−term matrix and the distribution of the selected three topics were analyzed. Two authors discussed the results and named these topics based on the terms generated under each topic. Table 2 presents the results of the identified three topics, the most popular terms within each topic, and the number and sample of posts under each topic.

## 4. Discussion

### 4.1. Emotional Aspect of Attitude towards Cyberbullying

In this study, the sentiment analysis showed that negative emotions dominated the Chinese public’s discussion of cyberbullying. The proportion of negative and positive emotions was 24.4% and 10.8%, respectively. Similarly, Tahamtan and Huang found that for English Twitter users, negative emotion words were more common than positive ones in posts related to cyberbullying [39]. Nevertheless, another cyberbullying study that analyzed English Twitter posts collected in 2016 showed that the English users’ attitude toward cyberbullying was largely neutral (43%, and “neutral” means not expressing any emotion), positive and negative attitudes toward cyberbullying were comparable (21%), and 16% had both positive and negative attitudes [32]. Our findings suggest that this is not the case among the Chinese public. More than half (51.7%) of Chinese Sina Weibo users have both emotional attitudes, but only 13.2% are neutral.

One possible explanation is the characteristics of the thinking mode in different cultures. Chinese users are more willing to take a stand on the topic of cyberbullying than English users, and such attitudes are often dialectical (i.e., positive and negative attitudes coexist). Influenced by the dialectical thinking mode in traditional Chinese culture [52], Chinese people tend to hold two attitudes towards a hot topic simultaneously [45]. The attitudes are characterized by cultural differences, and the emotional complexity (the co-occurrence of positive and negative affect) is more likely to occur in East Asian cultures with dialectical thinking than in North American cultures [53].

Another reason may be related to the rapid change of views on the Internet. While the current findings are similar to those of a 2019 survey [39], positive attitudes toward cyberbullying were significantly lower than those of a 2016 Twitter survey [32]. This shows that in recent years, the attitude of anti-cyberbullying has gradually taken root in people’s hearts. The amount of research on cyberbullying is increasing, and people’s understanding of the harm of cyberbullying is deepening. Therefore, the negative attitude is becoming stronger year by year. A study spanning 10 years (2005–2014) showed a significant increase in adult intervention in bullying and an overall decrease in bullying behavior over the years [54]. This also illustrates the growing negative attitude towards cyberbullying.

In the discussion of cyberbullying on Sina Weibo, sadness, anxiety, and anger increased in turn. Chinese people discuss cyberbullying mainly with anger. The finding was also found in a linguistic analysis of news about cyberbullying, where angry words accounted for the largest proportion of those used in media reports [55]. People often obtain information about cyberbullying from news reports on Sina Weibo; according to the framework theory of journalism [56], it is likely that the reported news highlighting anger has guided public opinion. Cyberbullying and emotions are connected [57,58], and anger is a common emotion in different roles (i.e., victim, perpetrator, and bystander) related to cyberbullying [59]. In addition, anxiety can be explained by the most-discussed topic of “critiques on cyberbullying and support for its victims”, because criticism means concern about its consequences and victims for the potential harm. We see very little sadness generated from the discussion, perhaps because sadness is primarily a victim-generated emotion [60]. The average percentage of victims among social media users is less than a third [61], so, in general, the public’s sadness is not very strong when cyberbullying is discussed.

### 4.2. Cognitive Aspect of Attitude towards Cyberbullying

The content analysis shows that the most common topics in the public discussion about cyberbullying are “critiques on cyberbullying and support for its victims”, “rational expressions of anger and celebrity worship”, and “calls for further control”. Corresponding to the results of sentiment analysis, all three topics reflected a strong attitude of anti-cyberbullying. This is also in line with the study that found that the public has a rather negative attitude towards cyberbullying in China [62]. Our findings suggest that people share information about the danger of cyberbullying and highly emphasize prevention.

The most discussed topic, “critiques on cyberbullying and support for its victims”, can be seen as the mainstream view of Weibo users currently. The media prefers to report on cyberbullying in terms of harm in order to gain public attention [55]. This may have sparked concern about victims and criticism towards cyberbullying. The second most-discussed topic, “rational expressions of anger and celebrity worship”, indicated that Weibo users emphasized the importance of rational online communication. Netizens often vent emotions by posting irrational comments about someone or something with insulting and inflammatory terms, which results in cyberbullying [63]. At present, the lack of rational behavior on the Internet is a crucial factor causing cyberbullying. More and more people attach importance to rational expression on social media [64]. This suggests that intervention policies can be developed by raising public awareness of rational action to prevent cyberbullying [10]. This method would be acceptable and popular with the public. Finally, the common topic, “calls for further control”, reflects the urgent needs of Weibo users. Cyber-regulators should pay special attention to the posts belonging to this topic, catch the public’s demands, and monitor the real-time changes in public opinion, which can provide a certain reference for the formulation of control policies.

Notably, our study identified three common topics, but other studies identified different categories of topics. McHugh et al. divided the discussion topics of cyberbullying on Twitter into six categories [32], and Tahamtan and Huang divided them into seven [39]. Their analyses showed that English Twitter users were concerned about cyberbullying towards celebrities, minors, and the general public; were less likely to directly express their likes and dislikes; and would pay a lot of attention to the control of cyberbullying.

Firstly, when it comes to cyberbullying, the public in both English-speaking and Chinese-speaking areas cares about both ordinary people and celebrities. These two objects happen to be common victims of cyberbullying [65], suggesting that the public in both English-speaking and Chinese-speaking areas may be more concerned about the victims of cyberbullying. Today, the Internet is accessible to everyone, so it is easy for ordinary people to become involved in cyberbullying and become the object of the discussion of cyberbullying. As for celebrities, they are public figures and their every move catches people’s special attention. Celebrities seem to be more likely to be victims of cyberbullying. The reason may be that celebrities are so far away from the public that the public is likely to tolerate cyberbullying on celebrities. There is a degree of a lack of empathy when it comes to celebrity cyberbullying, and some people feel that celebrities deserve more misfortune than non-celebrities [66].

Secondly, in contrast, the topic “critiques on cyberbullying and support for its victims” shows that when expressing support for victims, Twitter users rarely directly express likes and dislikes on cyberbullying, while Weibo users tend to express support or disapproval in stark terms. This direct appraisal may be a method of empathy expression. Several studies have found a significant negative correlation between levels of empathy and positive cyberbullying attitudes [62]. This study found that the Chinese public has a lower positive attitude towards cyberbullying, which may indicate more empathy expression.

Thirdly, Twitter users often call for control from schools, the media, laws, and so on, with a particular focus on protecting minors. Weibo users, however, are mainly calling for further control from the media and laws. Weibo and Twitter have become popular platforms for the public to express their views and suggestions towards the government. Hence, the calls are often proposed on Twitter and Weibo for authorities to control cyberbullying, such as requiring national legislation and using real-name registration in social media. A slight difference is that the public in the English-speaking areas is more interested in protecting minors than the Chinese public, which may be because teenagers in Europe and American countries experienced more cyberbullying.

### 4.3. Implications, Limitations, and Future Direction

In combination with big data technology, this study made full use of the social media corpus to analyze the emotional tendency and content of the Chinese public’s attitudes towards cyberbullying. This study overcame the limitations of traditional research methods, minimized the impact of social expectations, and truly reflected users’ attitudes toward cyberbullying. The optimized new method exceeded previous study paradigms of attitude, which not only considered positive and negative emotions, but also paid attention to the subdivided emotions, such as anger, anxiety, and sadness, to analyze the public’s attitude from multiple dimensions of emotion. In addition, a topic model was applied, and three discussion topics were automatically generated.

Some limitations of this study may affect the generalization of the results. First, in terms of data collection, considering the relationship between attitudes and time, our conclusions are inevitably affected by the timeline of data. Second, some noisy data cannot be removed due to technical limitations of the data cleaning step, and we are still actively looking for more efficient data processing techniques. Third, in terms of data analysis, the popular LIWC dictionary comes with word-based sentiment analysis, which may suffer from a lack of analytical accuracy. Fourth, this research adopted the unsupervised topic model and classified the data according to the intrinsic attribute relations, which is called the data-driven analysis. The current method is objective, but if we rely on automatic classification only, caution is needed when the results are interpreted.

The method of content analysis applied to cyberbullying on social media is of value, as well as for future research [67]. Some emerging text classification models employ advanced algorithms, such as neural network models, which may have a better predictive performance. In the future, we could build a supervised topic model based on the current results or take the latest algorithms to improve the text classification later. The current study only considered the textual features of the emotions and topics of the posts. Further research could consider adding other predictive variables, such as the way of thinking, cultural background, and participation in cyberbullying that we mentioned, to better understand how the public forms attitudes toward cyberbullying. A combination of multiple methods could also be a future trend, and studies could employ scales, experiments, and content analysis simultaneously to better explore cyberbullying. Our quantitative analysis could be a suitable starting point for the more qualitative survey using some traditional methods, such as focus groups and/or questionnaires.

## 5. Conclusions

To sum up, in this study, in China, Weibo users mainly held negative attitudes towards cyberbullying. Anger was the most common negative emotion when people talked about cyberbullying. Cyberbullying was sometimes discussed in relation to the emotion of sadness, but seldomly was discussed in relation to anxiousness. There were three main topics about cyberbullying on Weibo: critiques on cyberbullying and support for its victims, rational expressions of anger and celebrity worship, and calls for further control. Most people were well aware of the harm of cyberbullying, had a more rational view, and called for the cessation of cyberbullying. Given our current findings, we suggest that it is time to implement stricter control measures for cyberbullying. These measures could focus on the inclusion of programs to protect victims, educate about celebrity-worship behavior, and manage anger, as these are the elements that are of most concern to the public.

Our findings promote awareness of public attitudes toward cyberbullying. Negative public attitudes are a good prerequisite for promoting cyberbullying prevention and intervention measures [9]. The public’s strong anger and their needs for rational expressions of anger suggest advocating emotional management and the promotion of positive interaction on social media [57]. This study contributes to the comprehension of people’s demands and may provide guidance for targeted support. Interestingly, the work, although related to a different cultural and geographical context, allows us to find similarities even in the context of research in the Western world, on the phenomenon of cyberbullying, although the research here focused on social networks used exclusively in the Chinese context.

## Figures and Tables

**Table 1:** Examples of the five emotional linguistic categories [47].

Category	Examples
Positive emotion	love, nice, sweet
Negative emotion	hurt, ugly, nasty
Anxiety	worried, fearful, nervous
Anger	hate, kill, annoyed
Sadness	crying, grief, sad

**Table 2:** Identified topics, most popular terms and Weibo post samples.

Topic	Terms within Topics	Number of Posts	Weibo Post Samples
(1) Critiques on cyberbullying and support for its victims	like, fans, curse, why, star, diss, idol, harm, horrible, stand	24,975 (46.66%)	“…I’m not his fan. I just want to say something, and the fans shouldn’t curse a word... Cyberbullying is terrible. At least we’ve liked him so much before, we don’t want him to be depressed, right?”
(2) Rational expressions of anger and celebrity worship	Zhan Xiao 1, fans, resist, tip-off, stop, endorsement, history, time, star worshiping, oppose	14,640 (27.35%)	“…I don’t control the comments and the curses. It’s not that the fandom is not organized. We call for stopping his endorsement and business. We rationally consume instead of boosting his commerce. We want justice and fairness, resist Zhan Xiao and resist his fans. We hope there is no cyberbullying, no cyber manhunt. Zhan Xiao’s fans should stop... Although we have no capital and no organization, we will certainly not admit defeat…”
(3) Calls for further control	Weibo, snow melting agent 2, start a rumor, fans, evidence, real-name registration, comment, country, oppose, moral values	13,912 (25.99%)	“…Support opposing cyberbullying! The Internet has never been a place outside the law, and illegal acts such as cyberbullying are forbidden and not allowed. Whether it is for ordinary people or celebrities, I hope everyone will know the law and abide by the law”

1 “Zhan Xiao” is the name of a popular actor in China, who is highly discussed on the Internet. 2 The term “snow melting agent” came from a popular sentence in China. There is a buzzword on the Internet: No snowflake is innocent in an avalanche. It means when cyberbullying causes serious consequences on the victim, every perpetrator cannot escape from the responsibility. Therefore, “snowflake” refers to the cyberbullying perpetrators, and “snow melting agent” refers to people who want to prevent cyberbullying.

## Data Availability

The data presented in this study are available upon request from the authors.
